# Usage and preservation of Mizo traditional medicine by the people of Chungtlang Village, Mamit District, Mizoram

**DOI:** 10.5195/jmla.2024.1765

**Published:** 2024-07-01

**Authors:** Esther Lalruatpuii, R.K. Ngurtinkhuma, Samuel Lalruatfela, K.V. Reddy

**Affiliations:** 1 esther20chhangte.ec@gmail.com Research Scholar, Department of Library and Information Science, Mizoram University, Aizawl; 2 rkngur15@gmail.com, Professor, Department of Library and Information Science, Mizoram University, Aizawl; 3 samuruatfela94@gmail.com, Research Scholar, Department of Political Science, Mizoram University, Aizawl; 4 kvidyasagarr@gmail.com, Professor, Department of Political Science, Mizoram University, Aizawl

**Keywords:** Traditional, medicine, knowledge, infirmity, decoction, Mizo

## Abstract

Diversity, flexibility, easy accessibility, broad continuing acceptance in developing countries and increasing popularity in developed countries, relative low cost, low levels of technological input, relative low side effects, and growing economic importance are some of the positive features of traditional medicine. In rural India, traditional medicine continues to be the only available form of care. Many communities continue to treat patients using their old methods, unaffected by contemporary medical advancements. Due to their accessibility, affordability, and ease of use, tribal tribes prefer to utilize and consult their own traditional healers. These are likewise thought to be highly effective and without any adverse effects. This paper aims to identify various traditional medicines used for treating illness and infirmities, by taking accounts from the residents of Chungtlang village, Mamit District, Mizoram. The objective here lies in discovering traditional knowledge of medicinal plants and their uses for various infirmities.

## INTRODUCTION

A significant portion of the populace of a number of developing nations rely on a variety of goods based on traditional knowledge (TK) as vital sources of income, food, and health care. Traditional knowledge might belong to an individual, a group, or a whole society. Both industrialized and developing nations use traditional medicine extensively in their healthcare systems. In actuality, the majority of the people living in underdeveloped nations receive their health care from traditional medications and therapy systems since they are readily available and reasonably priced. Traditional medicines are a result of human medical practice in many regions of the world and are a reflection of human wisdom from thousands of years ago.

The Mizos of the state of Mizoram in north-eastern India use a variety of plants to heal a variety of diseases. Their practices are particular, and local elders or traditional healers frequently carry them out. A variety of plants used in their traditional medicine have obtained scientific approval for their usefulness and toxicity research. To be found and used, however, many more are required.

### What is Traditional Knowledge?

The term ‘traditional knowledge’ describes the ideas, inventions, and customs of indigenous peoples. Traditional knowledge is frequently passed down orally from generation to generation. It is developed through experience gathered over many years and is tailored to the local culture and environment. It typically belongs to everyone as a group and can be conveyed through myths, songs, folklore, proverbs, cultural values, beliefs, rituals, etc. It is also the origin of the traditional usage and management of lands, territories, and resources, including indigenous farming techniques that take care of the planet without depleting the resources. Indigenous peoples follow oral traditions, which have been practiced and passed down for millennia. These traditions include dances, paintings, sculptures, and other aesthetic manifestations [15].

Traditional knowledge is beneficial to contemporary business and agriculture as well as to individuals whose daily lives depend on it. The everyday routines and customs of indigenous peoples, as well as their in-depth knowledge of their ecosystems developed over many generations, serve as the foundation for traditional knowledge concerning land and species conservation, management, and restoration. It has the potential to significantly advance scientific, technical, and medical research, as demonstrated, among other things, by the pharmaceutical industry, and to solve the most critical global issues, including climate change, land management, and land conservation.

Traditional wisdom can also provide opportunities for ensuring food security for not just indigenous peoples but also for people everywhere. Numerous traditional methods of managing land and the environment have been shown to increase biodiversity locally and help keep ecosystems healthy. An important approach to conserve and preserve indigenous cultures and identities, lower illiteracy and school dropout rates, improve learning, save the environment, and promote welfare is through educational practices that incorporate indigenous traditional knowledge and languages [16].

### What is Traditional Medicine?

Traditional medicine is defined by the World Health Organisation as ‘health practices, approaches, knowledge, and beliefs incorporating plant, animal, and mineral based medicines, spiritual therapies, manual techniques, and exercises, applied separately or in combination to treat, diagnose, and prevent illnesses, or maintain well-being [13].’

Traditional medicine is the most ancient system of healthcare in existence, and it is used to treat and prevent both physical and mental ailments. In the past, different communities have created a number of practical healing techniques to treat a range of serious and life-threatening illnesses. Traditional medicine—also known as complementary and alternative medicine (CAM), ethnic medicine, or any other name—remains important in many nations today [18].

### Characteristics of Traditional Medicine

The word ‘ethno medicine’ refers to a traditional method of healing used by indigenous peoples that has to do with human health. It took hundreds of years of brave investigation and trial and error to learn which plants, animals, and minerals have therapeutic and palliative effects. This knowledge has been passed down from one generation to the next. Members of the community include the traditional herbalists. The prevalent illnesses of the populace are treated by the local healers in a home environment. Everyone is believed to be able to learn traditional medicine, and there are no formal educations or training requirements for using it.

In some families, nearly every member is familiar with one or more herbal medicines. The traditional healers are experts in specific fields of medicine. As a result, some doctors specialize in spiritual healing while others are experts in treating neurological illnesses, toxic stings, and setting broken bones. The effectiveness of herbal therapy is universally acknowledged by those who use it. Poor individuals in rural and urban areas rely on herbal cures since they are accessible to them. In fact, this is the sole accessible source of healthcare in isolated locations [11].

### Objectives and Need of the Study

The main objective of the study is to identify, discuss, and document the use of traditional Mizo medicines by the Mizos. Since time immemorial Mizos, even with the absence of scientific and medical know-how, have developed their own way of dealing with various illnesses and presumably offer treatment to those infirmities. Since this practice remained unpreserved and faces the threat of being vanished, preservation and proper documentation of these practices were the need of the hour.

In this contemporary advanced society, medical sciences offered humanity various antidotes to infirmities with advances in laboratory experimentation, with substantial negligence to traditional practices on medicines. Traditional medicines were deemed as superstitious and lack authenticity and reliability, however, one must not simply reject and neglect traditional medicinal knowledges because this knowledge is a product and result of centuries-old traditions.

### Scope and Limitation of the Study

The area under which the study is undertaken is limited to the residents of Chungtlang Village, Mamit District, Mizoram where the population is limited to 501 in 2022, as per village record put forth by Village Council. Various respondents across the village were asked of their knowledge on traditional Mizo medicines which were familiar to them. Accordingly, 15 native medicinal plants were observed. However, since these traditional medicines were prescribed without scientific and empirical evidence, and ultimately vested upon oral narratives, they lack credibility and authenticity.

### Methodology

The method utilized for collecting data involves the collection of secondary data from various sources like articles from recognized journals. Secondary data is collected by the author (s) observing the plants in the selected area under the guidance of the local people, with whom knowledge of these traditional medicines was procured.

## DISCUSSION

This research comprises lists of various traditional medicinal plants, some of which were peculiar to Mizos. Different plants were identified by their local name (with narration from the respondents in Mizo), their common name (in English), their botanical name, as well as their family in the botanical term.

**Table 1:** Traditional Mizo Medicines and Their Names

Sl No.	Local Name (in Mizo)	Common Name (in English)	Botanical Name	Family
1.	Tawkte	*Indian Night Shade*	*Solanum violaceum* Ortega	*Solanaceae (Potato family)*
2.	Saisiak	*White-Berry Bush*	*Flueggea virosa*	*Euphobiaceae*
3.	Tlangsam	*Siam weed*	*Eupatorium odoratum*	*Asteraceae*
4.	Hlingsi	*Chinese soapberry*	*Sapindus mukorossi*	*Sapindaceae*
5.	Phuihnam	*East Indian glory bower*	*Clerodendrum colebrookianum*	*Verbenaceae*
6.	Lambak	*Coinwort*	*Centella*	*asiatica Apiaceae (Carrot family)*
7.	Kelba-an	*Great plantain*	*Plantago major*	*Plantaginaceae*
8.	Sap Thei	*Passion fruit*	*Passiflora edulis* Sims	Assifloraceae (Passion flower)
9.	Sawhthing	*Ginger*	*ingiber officinale*	*Zingiberaceae*
10.	Aieng	*Turmeric*	*Curcuma longa*	*Zingiberaceae*
11.	Vailen Hlo	*Sticky daisy*	*Ageratum conyzoides*	*Asteraceae*
12.	Thingthupui	*Pithraj Tree*	*Aphanamixis polystachya*	*Meliaceae (Neem family)*
13.	Sekhupthur	*Lushai Begonia*	*Begonia lushaiensis*	*Begoniaceae (Begonia family)*
14.	Changkha	*Bitter Gourd*	*Momordica charantia*	Cucurbitaceae (Pumpkin) *family*
15.	Anhling	*Spiral Nightshade*	*Solanum spirale Solanaceae*	*(Potato family)*

Source: Websites

### Preparation and Uses of Mizo Traditional Medicines

#### 1. Tawkte (Indian Night Shade)

Its botanical name is *Solanum violaceum Ortega* from *Solanaceae (Potato family)*. The fruit juice is used topically to treat herpes. The green fruit was crushed and thus applied to the treatment of herpes. Additionally, the fruit is administered directly on hurting wounds.

**Figure 1:**
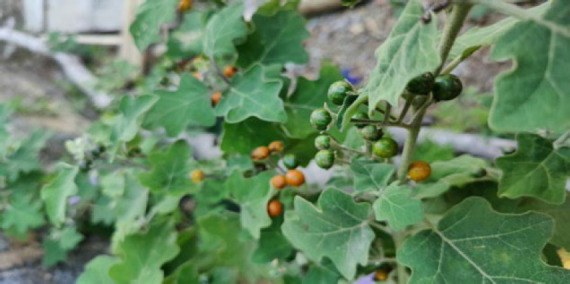
Leaves and fruits of *Solanum violaceum Ortega* in the wild.

#### 2. Saisiak (White-Berry Bush)

Its botanical name is *Flueggea virosa* from *Euphobiacease* family. The leaves are boiled and are used for taking baths for the treatment of Itching and Measles.

**Figure 2:**
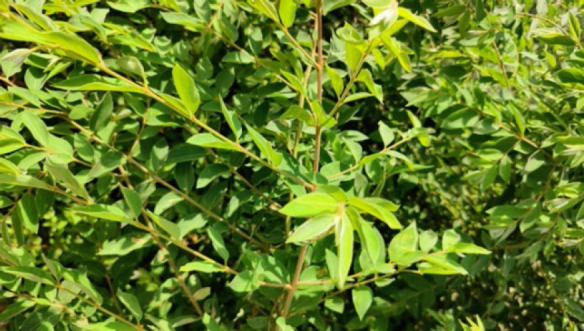
Leaves of *Flueggea virosa* in the wild.

#### 3. Tlangsam (Siam Weed)

Its botanical name is *Eupatorium odoratum* from *Asteraceae* family. The leaf juice is used topically as an anti-septic. The juice is also applied externally to remove pinworm from the anus.

**Figure 3:**
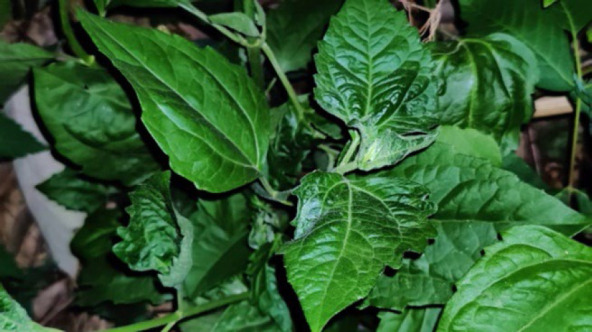
Leaves of *Eupatorium odoratum* in the wild.

#### 4. Hlingsi (Chinese soapberry)

Its botanical name is *Sapindus mukorossi* from *Sapindaceae* family. Consuming of the fruit pulp is used for the treatment of Pile disorder. Fruit juice is applied externally in mumps. One or two fruits are soaked in water overnight and the water is then used as a gargle for cough and tonsillitis. The fruit is a substitute for soap for the Mizo people in ancient times.

**Figure 4:**
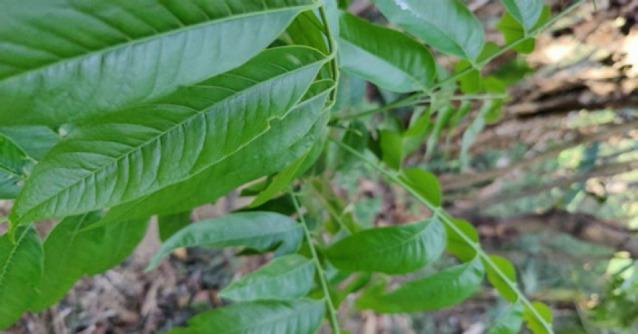
Leaves of *Sapindus mukorossi* in the wild.

#### 5. Phuihnam (East Indian glory bower)

Its botanical name is *Clerodendrum colebrookianum* from *Verbenaceae* family. Decoction of the leaves 2–3 times daily is given orally in the treatment of hypertension and also in diabetes. 5ml of the leaf juice is given orally and twice daily to treat colic in infants.

**Figure 5:**
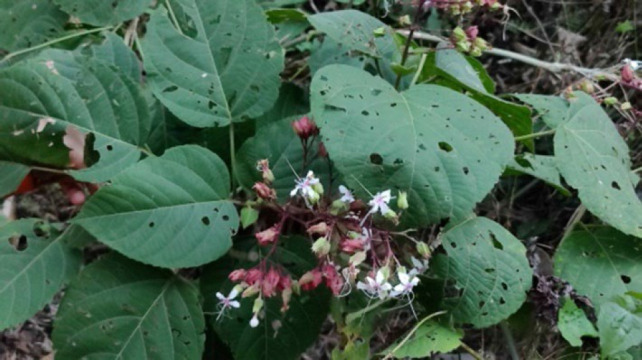
Leaves and flowers of *Clerodendrum colebrookianum* in the wild.

#### 6. Lambak (Coinwort):

Its botanical name is *Centella asiatica* from *Apiaceae* (Carrot family). It is said that *Centella asiatica* is effective for the treatment of skin disorders. It is also popularly used as a memory stimulator. The leaves are boiled and the water is taken for the remedy of asthma and eye problems. Decoction of the dried leaves is also used for controlling hypertension. The leaf juice is also used as a remedy for blood purification.

**Figure 6:**
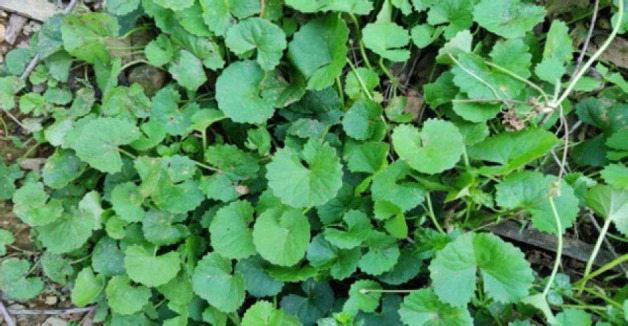
Leaves of *Centella asiatica* in the wild.

#### 7. Kelba-an (Green Plantain)

Its botanical name is *Plantago major* from *Plantaginaceae* family. The leaf juice is put in for earache and used locally for bee stings. To speed up the growth of new skin, the leaf decoction is applied directly to wounds and ulcers. It relieves toothaches and blisters on the gums. Decoction of the leaves is taken orally for the treatment of kidney and urinary problems, diabetes, malaria, and tuberculosis.

**Figure 7:**
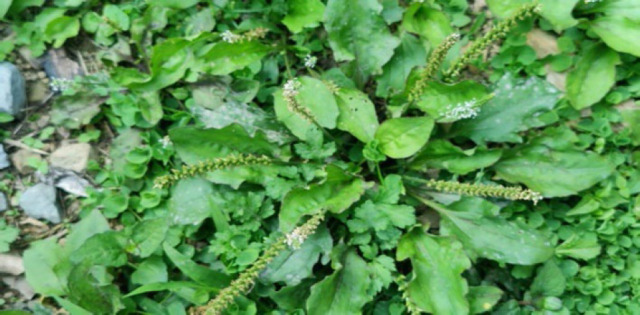
Leaves of *Plantago major* in the wild.

#### 8. Sapthei (Passion fruit)

Its botanical name is *Passiflora edulis Sims* from *Assifloraceae* (Passion flower). This plant is used for treating jaundice. For medicine, the fruit is orally consumed. In order to control high blood pressure, the teas of the dried leaves were also consumed.

**Figure 8:**
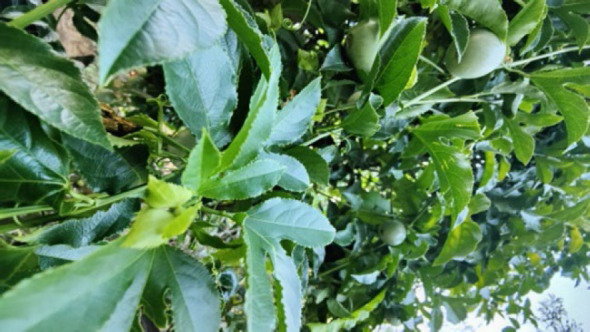
Leaves of *Passiflora edulis Sims* in the wild.

#### 9. Sawhthing (Ginger)

Its botanical name is *Zingiber officinale* from *Zingiberaceae* family. Extract i.e. ginger oil is used in cough & bronchitis; rhizome is roasted & eaten against throat pain, applied as a condiment; flowering bunches are sold in local markets as a vegetable.

**Figure 9:**
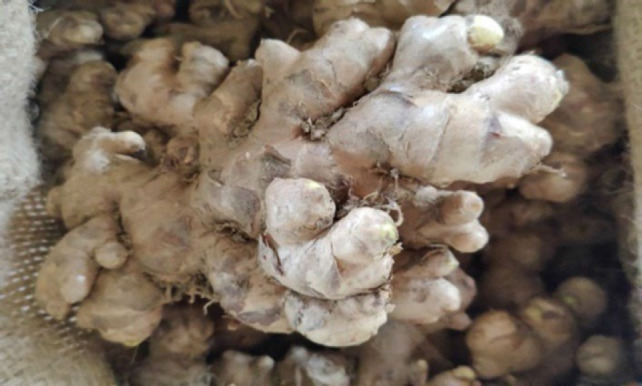
Shoot of *Zingiber officinale*.

#### 10. Aieng (Turmeric)

Its botanical name is *Curcuma longa* from *Zingiberaceae*family. Mainly by crushing the rhizome, the plant is used for the treatment of ulcer, diarrhea, asthma, and heart diseases. For the treatment of intestinal colic, the young shoot of this plant is also taken orally. Crushed fresh rhizome is applied immediately to swelling, cuts, and sprains as well.

**Figure 10:**
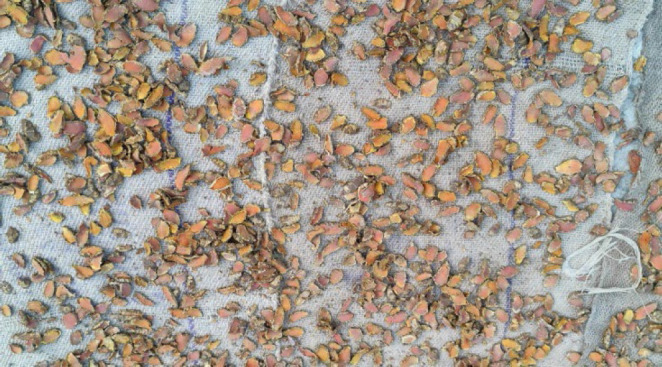
Dried *Curcuma longa* for further process.

#### 11. Vailen Hlo (Sticky daisy)

Its botanical name is *Ageratum conyzoides* from *Asteraceae* family. The plant crushed is taken orally for the treatment of cholera. On itches caused by insects or by hypersensitivity, the leaf juice is applied.

**Figure 11:**
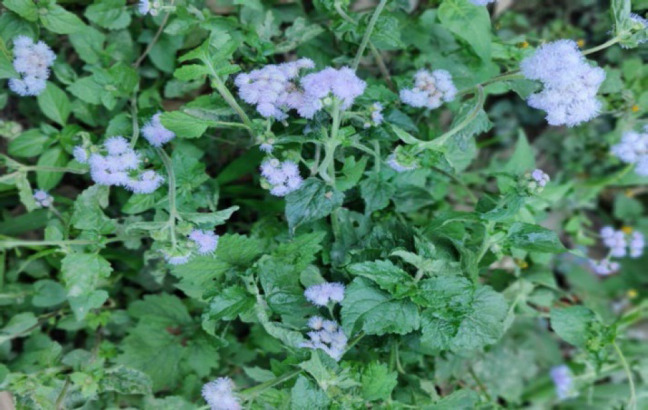
Leaves and flowers of *Ageratum conyzoides* in the wild.

#### 12. Thingthupui (Pithraj Tree)

Its botanical name is *Aphanamixis polystachya* from *Meliaceae* (Neem family). Decoction of the leaves is used for the treatment of dysentery, diarrhea, and hypertension.

**Figure 12:**
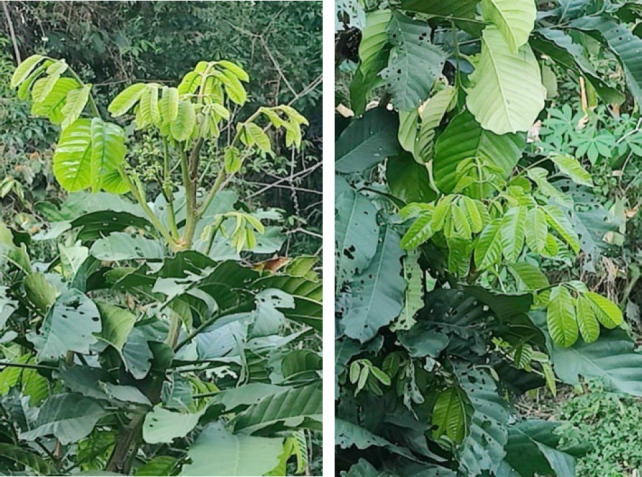
Young leaves of *Aphanamixis polystachya* in the wild.

#### 13. Sekhupthur (Lushai Begonia)

Its botanical name is *Begonia lushaiensis* from *Begoniaceae* (Begonia family). Decoction of the leaves and stems is taken orally for the treatment of dysentery, pile problem, diarrhoea, and malaria.

**Figure 13:**
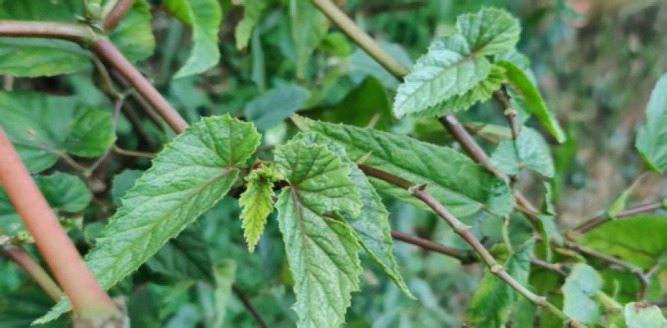
Young leaves of *Begonia lushaiensis* in the wild.

#### 14. Changkha (Bitter Gourd)

Its botanical name is *Momordica charantia* from *Cucurbitaceae* (Pumpkin family). The leaf juice is taken orally for the treatment of jaundice, and hypertension. The leaf juice is also used as nasal drops. It is also used externally and internally for dog bites.

**Figure 14:**
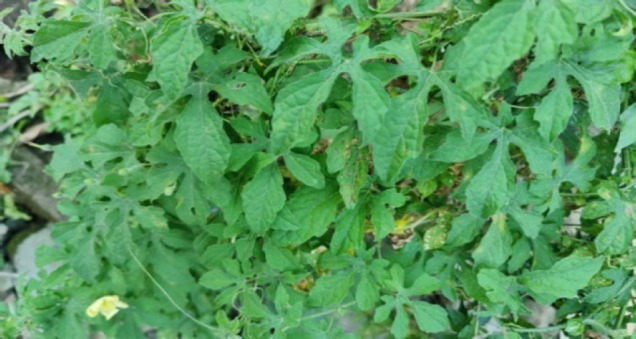
Leaves of *Momordica charantia* in the wild.

#### 15. Anhling (Spiral Nightshade)

Its botanical name is *Solanum spirale* from *Solanaceae* (Potato family). Decoction of the leaves is used for the treatment of urinary retention and kidney stone. Berry juice is used for the treatment of boils, ringworms, and to remove water leeches from both people and animals' noses.

**Figure 15:**
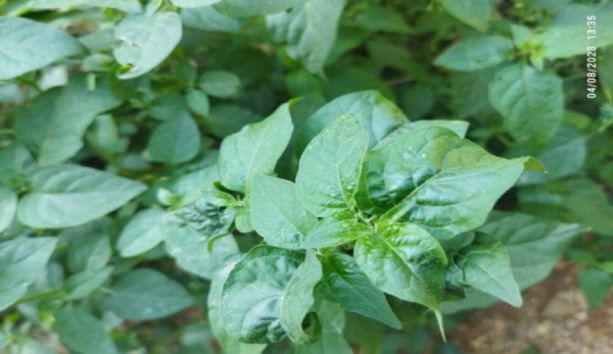
Leaves of *Solanum spirale* in the wild.

### Documentation of Traditional Medicine Practices in Library

The scientists and urban population are still unaware of a significant amount of knowledge gathered by the tribal members and peasants about herbal medicine. Numerous plant species found in rural areas are on the edge of extinction and are listed as vulnerable. Rural people are being displaced from their natural habitats as a result of deforestation, urbanisation, and industrialization, and their very expertise, particularly with regard to herbal medicines, is slowly vanishing.

Today, interest is growing from a wide range of disciplines, including ecology, soil science, health, medicine, botany, water resource management, and many more. This crucial area of concern has just lately been acknowledged by the Library and Information Science (LIS) community. Although indigenous knowledge is available in library and archive collections, LIS professionals frequently fail to contextualise it. Librarians expertly catalogue, digitise, and display material so that the general public can access it in favour of intellectual freedom. However, some of the main goals of libraries and other information services, such as freedom of speech, intellectual freedom, the dissemination of knowledge, research and learning, access to information, and the preservation of cultural heritage, are at right angles to indigenous claims for greater protection of Indigenous Knowledge systems and cultural material [17]. There is a lot that LIS professionals can accomplish in the overall management of Indigenous Knowledge to make the documentation and distribution of Indigenous Knowledge a reality [2].

Mabawonku (2002) explains that information professionals have important responsibilities to play as development agents in discovering, gathering, interpreting, sharing, and conserving Indigenous Knowledge. Because of its stable position both within the community and within the government framework through which it is formed, the public library, for example, has been an ideal anchor partner in Indigenous Knowledge system related activities [4]. According to the International Federation of Library Association (2003), libraries can assist in gathering, preserving, and disseminating indigenous and local traditional knowledge as well as educating both non-indigenous and indigenous peoples about the value, contribution, and importance of indigenous knowledge [6].

## CONCLUSION

Utilizing native flora for healing and implementing practices that improve community health is part of traditional medicine. The inclusion of information related to traditional knowledge in a common language and in an accessible manner has tremendously aided efforts to harness and develop it for use in the future. Libraries may be very helpful in keeping traditional medicines alive. Librarians may assemble traditional medical knowledge in both book and non-book formats using their experience in information management. Libraries should cooperate and collaborate closely with indigenous practitioners who are custodians of unpublished records and who are also within the purview of Library and Information Science of unwritten information in order to close the gap between the practices of information management by non-professionals who had focused primarily on unpublished information resources.
